# The effect of music in gynaecological office procedures on pain, anxiety and satisfaction: a randomized controlled trial

**DOI:** 10.1186/s10397-017-1016-2

**Published:** 2017-08-09

**Authors:** N. Mak, I. M. A. Reinders, S. A. Slockers, E. H. M. N. Westen, J. W. M. Maas, M. Y. Bongers

**Affiliations:** 1 Department of Obstetrics and Gynaecology, Máxima Medical Centre, Veldhoven, The Netherlands; 2 Department of Obstetrics and Gynaecology, VieCuri Medical Centre, Venlo, The Netherlands; 3 Department of Obstetrics and Gynaecology, Rode Kruis Hospital, Beverwijk, The Netherlands; 4 Department of Obstetrics and Gynaecology, GROW—School for Oncology and Developmental Biology, Maastricht University Medical Centre, Maastricht, The Netherlands

**Keywords:** Pain, Anxiety, Music, Office procedures, Hysteroscopy, Colposcopy

## Abstract

**Background:**

Pain can interfere with office procedures in gynaecology. The aim of this study is to measure the positive effect of music in gynaecological office procedures.

**Methods:**

A randomized controlled trial was performed between October 2014 and January 2016. Women scheduled for an office hysteroscopy or colposcopy were eligible for randomization in the music group or control group. Stratification for hysteroscopy and colposcopy took place. The primary outcome is patients’ level of pain during the procedure measured by the visual analogue scale (VAS). Secondary outcomes include patients’ level of pain after the procedure, anxiety and satisfaction of patient and doctor.

**Results:**

No positive effect of music on patients’ perception of pain during the procedure was measured, neither for the hysteroscopy group (57 mm vs. 52 mm) nor for the colposcopy group (32 mm vs. 32 mm). Secondary outcomes were also similar for both groups.

**Conclusions:**

This study showed no positive effect of music on patients’ level of pain, anxiety or satisfaction of patient or doctor for office hysteroscopy and colposcopy. We believe a multimodal approach has to be used to decrease patient distress in terms of pain and anxiety, with or without music.

**Trial registration:**

Dutch Trial Register, NTR4924

## Background

Today, office procedures in gynaecology are widely used to diagnose and directly treat gynaecological abnormalities [1–3]. However, pain and anxiety remain problems that may impede the procedure and can contribute to a negative experience for the patient [4–8].

Listening to music could be an easy and non-invasive way to decrease pain and anxiety. However, the literature is not clear about the efficacy of music therapy. Music for pain relief of any type was previously examined in a review including 31 studies. The studies showed a high variation in the results. Pooled data demonstrated a significant reduction of 0.4 points on a 0–10 scale, which is of doubtful clinical importance [9]. Research on this topic in gynaecology is also not conclusive. The meta-analysis of Wang et al. suggested a positive effect of music regarding pain, anxiety and satisfaction for patients undergoing endoscopic surgery. For patients undergoing colposcopy, no effect was found [10]. This result is the sum of two randomized controlled trials with contradictory results regarding the impact of music in office colposcopy, with no effect versus an almost 2 point decrease in pain measured by the VAS (0–10) in favour of music therapy [7, 11, 12]. Only one article could be found on the effect of music during office hysteroscopy. Angioli et al. showed a positive effect of the use of music with a reduction of pain and anxiety [13].

The effect of music in gynaecological office procedures on satisfaction of patients is less frequently examined. Danhauer et al. found no effect [11]. Other studies in office gynaecology did not examine the efficacy of music on satisfaction of patients [12, 13]. The satisfaction of the doctor is not described in any of these articles [11–13]. However, music can have a negative influence on task performance and level of irritation of the surgeon in laparoscopic surgery [14, 15]. Therefore, this satisfaction of the doctor should not be ignored.

Previous research on the effect of music in gynaecological office procedures was not blinded for patients or doctors, meaning there was a risk of bias [11–13]. Moreover, other interventions to decrease the patient’s discomfort, such as verbal communication between patient and doctor or nurse, are not mentioned or are excluded in previous studies [11–13]. Positive interactions between patient and doctor or nurse may interact with pain and anxiety and such interaction is often used in daily practice. The use of local anaesthetics, the use of information leaflets and the use of videoscopy are all methods used to improve patients’ experience [3, 6, 7, 16].

We can conclude from previous research that a large discrepancy exists in the efficacy of music in the reduction of pain and anxiety. Research on the effect of music on the satisfaction of patient and doctor is rare. Previous research is possibly biased and does not answer the question of whether music is beneficial for patients and doctors in daily practice. The aim of this study is to demonstrate the complementary value of music in gynaecological office procedures on patients’ level of pain, anxiety and satisfaction during and after the procedure in daily practice. The experience of the doctor will be evaluated as well.

## Methods

### Trial design

Between October 2014 and July 2016, a single-blind prospective randomized controlled superiority trial was performed at the Department of Obstetrics and Gynaecology in the Máxima Medical Centre in Veldhoven, the Netherlands. The trial was approved by the Medical Ethics Committee of the hospital (Study number 2014–28) and was registered in the Dutch Trial Register under trial ID number NTR4924.

### Participants

All patients who referred to the outpatient clinic for a hysteroscopy or colposcopy were considered for inclusion. Inclusion criteria were Dutch-speaking women, of at least 18 years of age, planned for an office hysteroscopy or colposcopy with biopsy or large loop excision of the transformation zone (LLETZ). Exclusion criteria were hearing impairments, blindness and known anatomical characteristics that may make performing the office procedure more difficult (e.g., cervical conization, Manchester Fothergill).

### Outcomes

The primary outcome was the experience of pain during the procedure, measured with the VAS on a 0–100 mm scale. The measurement took place during biopsy or LLETZ in the group of women undergoing colposcopy or during the passage of the internal ostium in the group of women undergoing hysteroscopy. Secondary outcomes were heart rate and anxiety during the procedure, pain after the procedure and satisfaction of patient and doctor. The heart rate was measured by using a pulse oximeter. The highest heart rate was used which was measured at the same time as the VAS during the procedure.

Anxiety of the patient was measured using the validated Dutch version of the State-Trait Anxiety Inventory (STAI) before and after the procedure. State anxiety and trait anxiety were both assessed by 20 items with scores ranging from 20 to 80, with higher scores indicating greater levels of anxiety. Satisfaction of patient and doctor was described using a scale of 1 to 5. In addition, the participants were asked if they would recommend the procedure in this setting to a friend. The doctor was asked if he or she would like to repeat the procedure in the same setting. Further, if applicable, the doctor was asked for the level of irritation regarding the music. Data and scores were reported in a case report form (CRF) and subsequently imported in the database by one person and controlled by another person. Excel was used as database.

### Procedure

The researchers informed the patients who met the inclusion criteria in advance of their hospital visit. The eligible participants were told that they would participate in a study of pain relief during office procedures. In order to perform single-blind testing, they were not informed about the role of music. After giving informed consent, the following information was collected from all participants before the procedure: age, height, weight, drug use, use of painkillers before the procedure, parity, intensity of dysmenorrhoea and expected pain of the procedure both measured by the VAS. Participants were asked to arrive 15 min before their appointment to fill out the questionnaire with baseline characteristics and the STAI. Furthermore, the participant’s heart rate was measured before the procedure.

The researchers randomized participants to the music group or control group. Stratification for hysteroscopy and colposcopy took place. Sealed numbered opaque envelopes were used for randomization. Participants who were randomized for the music group could choose between three types of music: pop, classical music and spa music. An iPod with speakers was used to play the music instead of headphones to maintain a good communication. The volume of the speakers could be adjusted by the doctor or researchers in a way so that the music was audible without disturbing the interaction between the participant and doctor or nurse. A gynaecologist or a resident performed the procedure. The experience of pain in VAS and participant’s heart rate were measured during the procedure.

To determine whether or not music is beneficial for patients and doctors in daily practice, other contemporary interventions to decrease patients’ discomfort remained unchanged with respect to the standard procedure. These include the use of information leaflets, the advice to use a painkiller before the procedure, the communication between patient and doctor, the emotional support by a nurse and the use of videoscopy during the procedure. A cervical block for patients undergoing a colposcopy was used if indicated according to the doctor.

After the procedure, participants were asked again to fill out a questionnaire regarding their level of satisfaction and the STAI. The doctor was asked for the degree of difficulty of the procedure. To achieve a smooth implementation of the study without influencing the standard procedure, a pilot study with ten participants was conducted before the start of this study to train the staff.

### Statistical methods

Based on the previous research, a decrease of 20 mm on the VAS scale was expected when using music during the procedure [12, 13]. A sample size of 38 participants for each arm was calculated for both hysteroscopy and colposcopy based on the power analysis with a power of 0.90, a 5% significance level and an expected loss to follow-up of 5%.

The Shapiro-Wilk test was used to test the normality of the data. Depending on this result, the *t* test or the Mann-Whitney *U* test was used. Categorical data were tested using the chi-square test. If there was a statistical significant difference in base characteristics between the music group and the control group, linear regression was used to test for confounding. In case of confounding, the primary outcome was calculated with correction of these variables by linear regression. For all outcomes, the intention-to-treat analysis was used. In addition, a per-protocol analysis was performed for the primary outcome. All statistical analyses were performed using IBM SPSS Statistics for Windows (version 21.0, Armonk: NY, IBM Corp).

## Results

### Hysteroscopy

**Fig. 1:**
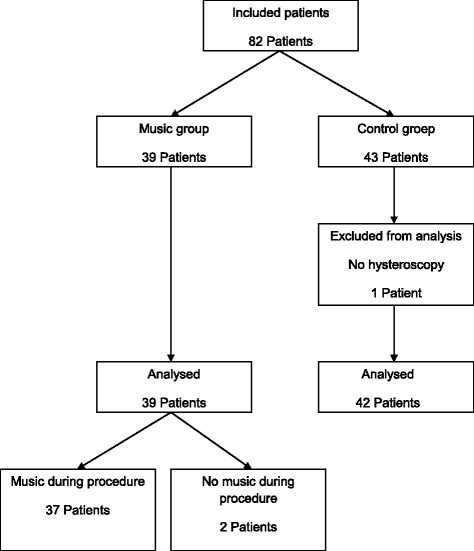
Hysteroscopy: flow chart patient inclusion

Eighty-two participants were included, 39 participants in the music group and 43 participants in the control group. One participant in the control group received a saline infusion sonohysterography (SIS) only and was excluded from further analyses. Thus, 81 participants (39 in the music group and 42 in the control group) were considered for the statistical analyses. Two participants from the music group did not receive music during the passage of the internal ostium of the cervix, due to a technical problem with the iPod (Fig. 1).

Baseline characteristics are shown in Table 1. These characteristics were similar for both groups. No statistical significance was found for pain during the procedure between the music group and the control group (57.1 (25.7) mm vs. 51.6 (27.1) mm, *p* = .382). Secondary outcomes were also similar, including heart rate and anxiety during the procedure, pain after the procedure and satisfaction of patient and doctor (*p* > .05) (Table 2). No complications occurred.

**Table 1:** Hysteroscopy: patient characteristics

	Music group *N* = 39	Control group *N* = 42
Age (y)	45.4 (13.2)	45.2 (15.0)
Height (m)	1.67 (0.07)	1.66 (0.07)
Weight (kg)	77.3 (20.7)	73.2 (14.5)
Body mass index	27.6 (7.6)	26.7 (6.4)
Dysmenorrhea (mm VAS)	38.7 (31.4)	41.0 (26.7)
Expected pain (mm VAS)	51.2 (21.3)	54.6 (22.9)
Heart rate before procedure (bpm)	80.4 (11.1)	78.6 (13.2)
Use of a painkiller (%)	82	88
Difficulty of procedure (%)		
Very easy	27	37
Easy	40	23
Normal	19	34
Difficult	11	3
Very difficult	3	3
Intervention (%)		
Diagnostic	23	31
Biopsies	23	21
Therapeutic	54	48
Diameter 5.5 mm Hysteroscope (%)	67	74
STAI 1 score before procedure	40.7 (13.0)	42.6 (12.5)
STAI 2 score	34.0 (8.0)	36.0 (10.5)

Data are expressed as mean (SD) or percentage

**Table 2:** Hysteroscopy: results

	Music group *N* = 39	Control group *N* = 42	*P* value
Pain during procedure (mm VAS)	57.1 (25.7)	51.6 (27.1)	0.382
Pain after procedure (mm VAS)	29.2 (25.9)	32.2 (27.8)	0.715
Heart rate during procedure (bpm)	82.6 (14.0)	83.3 (13.7)	0.833
Patient recommend a friend (%)	97	93	0.617
Patient satisfaction (%)			0.958
Very satisfied	59	62	
Satisfied	31	31	
Normal	8	5	
Dissatisfied	2	2	
Very dissatisfied	0	0	
Doctor refuses same procedure in same setting for this patient (%)	5	2	0.610
Satisfaction doctor (%)			0.165
Very satisfied	50	60	
Satisfied	29	20	
Normal	13	5	
Dissatisfied	0	10	
Very dissatisfied	8	5	
Complications (%)	0	0	NS
STAI 1 score after procedure	34.1 (8.6)	35.9 (9.6)	0.491
STAI 1 score difference	6.3 (12.8)	5.7 (10.9)	0.820

Data are expressed as mean (SD) or percentage

*NS* not significant

In three cases (8%) in the music group, the doctor was (very) dissatisfied. One doctor reported that this was caused by the music, which was not a genre he or she enjoys. In another procedure, the doctor mentioned the dissatisfaction was correlated with the difficulty of the procedure. The reason for the last case was not reported. In two cases (5%), the doctor in the music group mentioned that he or she did not want to repeat the procedure in the same setting. The disturbing music was the reason for one of these two cases. The most popular music choice in the music group was pop music (58%), followed by classical music (21%) and spa music (21%).

Besides an intention-to-treat analysis, a per-protocol analysis was performed only for the primary outcome because two participants from the control group did not receive music during the passage of the internal ostium. Again, no difference was found (59.2 (24.3) mm vs. 50.0 (27.7) mm, *p* = .154).

### Colposcopy

**Fig. 2:**
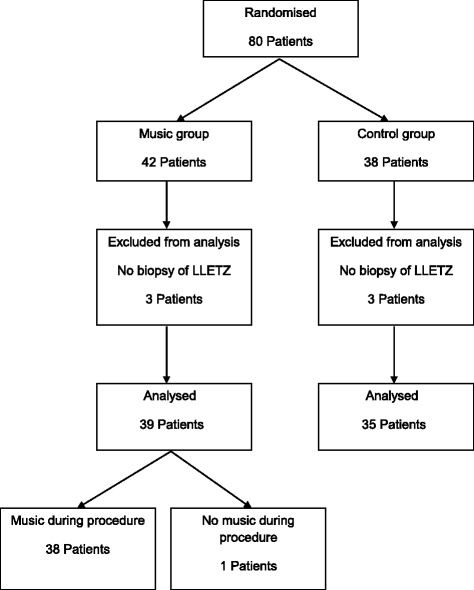
Colposcopy: flow chart patient inclusion

Eighty participants were included, 42 participants in the music group and 38 participants in the control group. In each group, 3 participants did not meet the inclusion criteria because no biopsy or LLETZ was performed during colposcopy. These participants were excluded from further analyses. Therefore, 74 participants (39 music group and 35 control group) were considered for statistical analyses. One participant of the music group refused music during the procedure (Fig. 2).

Baseline characteristics are shown in Table 3. A significant difference between the groups was found for dysmenorrhoea (24.1 (24.8) mm vs. 38.2 (22.9) mm, *p* = .013) and the performance of a cervical block (72 vs. 47%, *p* = .031). For all other characteristics, no difference was found (*p* > .05). No significant difference was found for pain during the procedure between the music group and control group (32.4 (24.3) mm vs. 31.6 (27.3) mm, *p* = .826). Secondary outcomes were also similar, including heart rate and anxiety during the procedure, pain after the procedure and satisfaction of the patient and doctor (*p* > .05) (Table 4). No complications occurred.

**Table 3:** Colposcopy: patient characteristics

	Music group *N* = 39	Control group *N* = 35
Age (y)	38.8 (8.3)	38.9 (10.7)
Height (m)	1.69 (0.06)	1.70 (0.06)
Weight (kg)	69.0 (13.3)	69.6 (17.8)
Body mass index	24.0 (4.1)	24.1 (6.1)
Dysmenorrhea (mm VAS)	24.1 (24.8)	38.2 (22.9)
Expected pain (mm VAS)	43.1 (23.9)	49.4 (22.1)
Heart rate before procedure (bpm)	78.2 (15.1)	82.1 (14.8)
Use of a painkiller (%)	8	9
Use of cervical block (%)	72	47
Pap smear score (PAP) (%)		
PAP 2	29	26
PAP 3a	48	48
PAP 3b	23	26
Colposcopic impression (%)		
Normal	6	7
Low grade	47	64
High grade	44	29
Carcinoma	3	0
Difficulity of procedure (%)		
Very easy	47	65
Easy	29	26
Normal	18	0
Difficult	3	6
Very difficult	3	3
Intervention (%)		
Cold biopsy	28	54
Hot biopsy	3	0
LLETZ	69	46
STAI 1 score before procedure	42.1 (12.3)	42.6 (8.7)
STAI 2 score	34.9 (10.1)	34.3 (7.9)

Data are expressed as mean (SD) or percentage

**Table 4:** Colposcopy: results

	Music group *N* = 39	Control group *N* = 35	*P* value
Pain during procedure (mm VAS)	32.4 (24.3)	31.6 (27.3)	0.826
Pain after procedure (mm VAS)	23.6 (21.5)	27.6 (25.6)	0.637
Heart rate during procedure (bpm)	82.4 (16.1)	82.7 (15.1)	0.929
Patient recommends a friend (%)	95	88	0.408
Patient satisfaction (%)			0.571
Very satisfied	77	79	
Satisfied	15	15	
Normal	3	3	
Dissatisfied	5	0	
Very dissatisfied	0	3	
Doctor refuses same procedure in same setting for this patient (%)	5	3	1.000
Satisfaction doctor (%)			0.769
Very satisfied	74	79	
Satisfied	18	15	
Normal	5	6	
Dissatisfied	3	0	
Very dissatisfied	0	0	
Complications (%)	0	0	NS
STAI 1 score after procedure	38.4 (15.3)	35.5 (9.6)	0.584
STAI 1 score difference	3.8 (14.4)	7.5 (10.2)	0.463

Data are expressed as mean (SD) or percentage

*NS* not significant

In five cases in the music group (12%), the doctor noticed he or she was disturbed by the music during the procedure. In three of these cases, the volume of the music was too loud; in one case, the music was not of the genre preferred by the doctor; and in the last case, no explanation was given. In one case in the music group, the doctor was dissatisfied without mentioning a reason (3%). The most popular music genre chosen in the music group was pop music (67%), followed by classical music (18%) and spa music (15%).

In addition to the intention-to-treat analysis, a per-protocol analysis was performed only for the primary outcome because one participant refused music. Still no difference was found in pain between the groups (33.3 (24.0) mm vs. 30.7 (27.4) mm, *p* = .579). Dysmenorrhoea and performance of a cervical block were both different between the groups (Table 3). After performing a linear regression, we concluded that both variables are confounders for the primary outcome. Correction of these variables for pain during the procedure resulted in the same conclusion, i.e. no statistical significance between the two groups (*p* = .806). A per-protocol analysis with correction for these two confounders showed the same result (*p* = .563).

## Discussion

### Main findings

The aim of this study was to measure the additional effect of music in gynaecological office procedures on patients’ level of pain, anxiety and satisfaction during and after the procedure in daily practice. The experience of the doctor was evaluated as well. We found no positive effect of music, neither in hysteroscopy nor in colposcopy.

### Strength and limitations

To our knowledge, this is the first randomized controlled trial investigating the effect of music in gynaecological office procedures taking into account the opinion of the doctor. Moreover, we explored the additional effect of music in daily practice. Methods that were already used to improve patients’ experience remained unchanged with respect to the standard procedure to increase external validity. Another asset of this study is its use of single-blind testing. The participants in this study were not informed about the role of music in this study; they were only informed about the goal to improve patients’ experience during office procedures in a non-invasive manner with a controlled trial. This is unique in comparison to other studies.

A limitation of this study is that waiting time and duration of the procedure were not examined. Waiting time can possibly change the anxiety and pain level of the patient and prolonged duration of the procedure can increase the dissatisfaction of the patient and the doctor. Another limitation is the difference in experience between the doctors. In both groups, hysteroscopy and colposcopy, the doctors consisted of both gynaecologists and residents. According to the literature, pain scores can be lower when an experienced doctor performs the procedure [17]. iPod speakers were used to play the music which prevented double-blind testing. However, during the pilot study, headphones turned out to impede the interaction between patient and doctor. For this reason, headphones were waived.

### Interpretation

Despite randomization, we found a difference between dysmenorrhoea and the use of a cervical block between the groups in the patients receiving a colposcopy. The women in the music group had less dysmenorrhoea, but more of them received cervical anaesthesia (Table 3). Significantly, less dysmenorrhoea could imply a higher pain threshold in that group which may confound the primary outcome. The difference in cervical anaesthesia could be explained by the difference in intervention between the groups (*p* = .056). Women who underwent cold biopsies did not receive a cervical block in contrast with electrical biopsies and LLETZ. The cervical block given in this trial consists of an anaesthetic (articaine) and a vasoconstrictor (epinephrine). According to Gajjar et al., receiving local anaesthetics and a vasoconstrictor could possibly reduce pain experience in women undergoing colposcopy. Therefore, the difference in dysmenorrhoea and the use of a cervical block between the groups is relevant. For this reason, a correction was performed for these confounders. Still, no difference was found between the music group and control group. Thus, the result remained unchanged.

Previous research in music for pain relief showed a large difference in results with high heterogeneity in studies as described in the systematic review of Cepeda et al. A positive effect of music in gynaecological office procedures was found in randomized controlled trials performed by Angioli et al. and Chan et al. However, another randomized controlled trial by Danhauer et al. found no difference between the music group and the control group for pain, anxiety or satisfaction. These results are similar to the results in this current trial. Danhauer et al. suggest that their results are probably different from the results of the two previously mentioned trials because of the limited choice of five music genres, the number of physicians and the difficulty in hearing what the doctor was saying because of the headphones. However, according to a systematic review, the decline in pain intensity is similar in studies wherein patients selected the type of music and in those wherein patients did not select their music [9]. Instead of the headphones used in the trial of Danhauer et al., iPods with speakers were used in our study, giving the same result.

The potential positive effect of music may have been overpowered by the multimodal approach in our study. The use of information leaflets, analgesics, the interaction between patient and doctor, a nurse to offer emotional support and the use of videoscopy are all used in daily practice. For that reason, they remained unchanged with respect to the standard procedure in this trial. Information leaflets increase the patient’s knowledge and therefore could improve the patient’s experience [7]. The value of oral analgesics is limited [3, 18], but local anaesthesia could be effective at achieving pain relief [3]. A monitor for videocolposcopy, allowing the patient to view the procedure, reduces patient anxiety and pain during routine colposcopic examination [16]. Finally, active emotional support can reduce pain [19].

Another explanation for our results, which is possibly associated with the multimodal approach described above, is the relatively low pain score in our trial. The control group in the colposcopy group showed lower scores in comparison with the trials of Chan et al. and Danhauer et al., namely 31.6 in this trial versus 50.3 and 51.7 in the other trials. The power analysis and expected pain reduction in this trial were based on these results from previous trials. Moreover, a score of VAS 40 is frequently used in the literature as a pain threshold [17, 20, 21]. Therefore, with an initial pain score lower than 40, the clinical relevance of pain relief is doubtful. Thus, we believe that our multimodal approach already greatly improves patients’ experience and possibly hereby camouflages the potential effect of music.

We found no difference in the satisfaction of the doctors between the music group and the control group for both hysteroscopy and colposcopy. However, some doctors mentioned that they were disturbed by the music; one case in the hysteroscopy group (3%) and five cases in the colposcopy group (12%). The difference between the two groups can be explained by the different doctors performing a hysteroscopy or a colposcopy. Despite the fact that the volume could be adjusted, the reasons they mentioned for their irritation were the volume of the music and the fact that it was not the kind of music they enjoy. Therefore, perhaps the use of more neutral music set at a lower volume would satisfy these doctors. Unfortunately, we did not examine the music preferences of the doctors.

## Conclusion

In conclusion, our study showed no positive effect of music regarding pain, anxiety or satisfaction for office hysteroscopy and colposcopy. We believe a multimodal approach should be used to decrease patient distress in terms of pain and anxiety, with or without music.
